# A rare case of primary inverted papilloma of the middle ear

**DOI:** 10.11604/pamj.2019.33.49.18065

**Published:** 2019-05-22

**Authors:** Mehdi Hasnaoui, Mohamed Masmoudi, Nouha Ben Abdeljelil, Nouha Ben Hmida, Nabil Driss

**Affiliations:** 1 Department of Otolaryngology-Head and Neck Surgery, Tahar Sfar Hospital, Mahdia, Tunisia; 2 Department of Pathology, Fattouma Bourguiba Hospital, Monastir, Tunisia

**Keywords:** Inverted papilloma, middle ear, surgery

## Abstract

Inverted papilloma (IP) of the middle ear as a primary lesion or as an extension of a sinonasal papilloma, is extremely rare. Only 23 cases of primary inverted papilloma of the middle ear have been reported in the literature. They are locally aggressive tumours, with a high rate of recurrence and associated malignancy. We present a rare case of a 59-year-old man presenting with unilateral otorrhoea, tinnitus, and hearing loss. Otoscopy revealed posterior perforation filled by irregular budding neoformation. The temporal CT scan showed tissue filling the tympanic cavity as well as the mastoid antrum without bone lysis. The patient underwent limited tympanoplasty. An intraoperative biopsy of polypoid tissue filling the tymapanic cavity was made and histopathology showed an IP. A recurrence occurred 4 months after surgery. We performed open tympanoplasty allowing complete resection of the lesions, with no recurrence after a follow-up of 30 months.

## Introduction

Inverted Papilloma (IP) is a benign tumor that develops primarily in the sinonasal tract. Localization in the middle ear, regardless of primary or extension of a sinonasal IP, is exceptional. Only 23 cases of primary inverted papilloma have been reported in the literature [1-6]. From an observation and review of literature, we propose to specify the diagnostic, therapeutic and evolutionary features of this condition.

## Patient and observation

**Figure 1:**
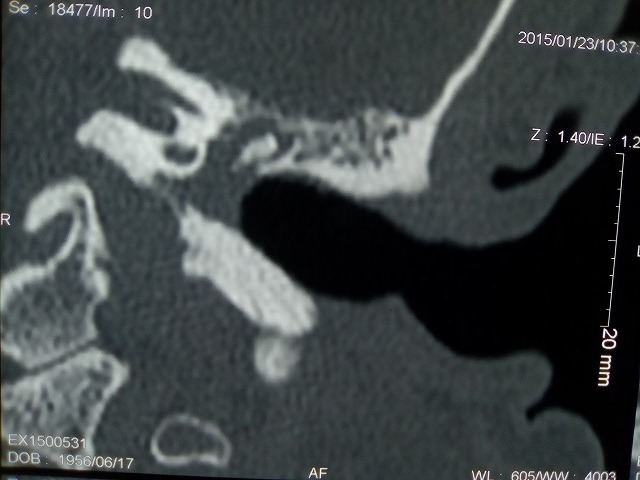
Coronal view of temporal CT showed complete opacification of the middle ear and mastoid indicating a soft tissue mass

**Figure 2:**
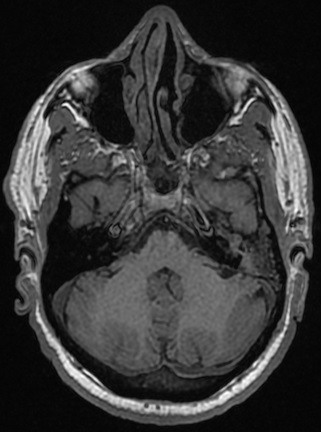
Magnetic resonance imaging (MRI) revealed a tissue process occupying the left middle ear in T1 isosignal

**Figure 3:**
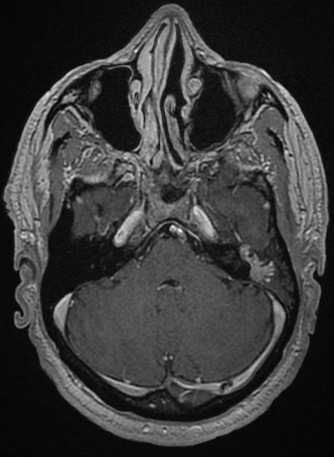
T1 MRI post-contrast demonstrating mild enhancement of the lesion in the left middle ear

**Figure 4:**
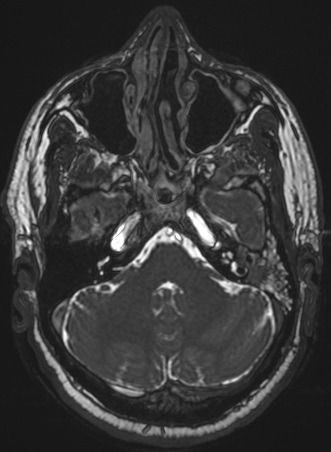
Magnetic resonance imaging (MRI) revealed a tissue process occupying the left middle ear in T2 isosignal

**Figure 5:**
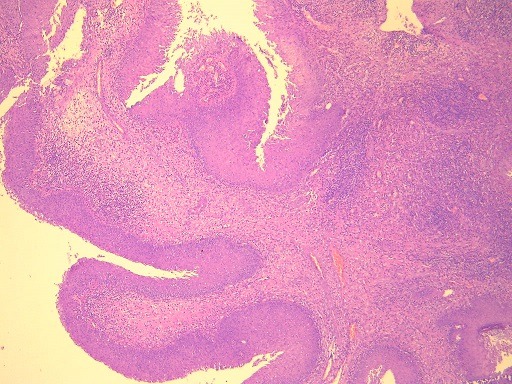
Microscope image showing an inverted papilloma: papillomatous proliferation of the squamous epithelium with endophytic growth pattern (HE x 40)

**Figure 6:**
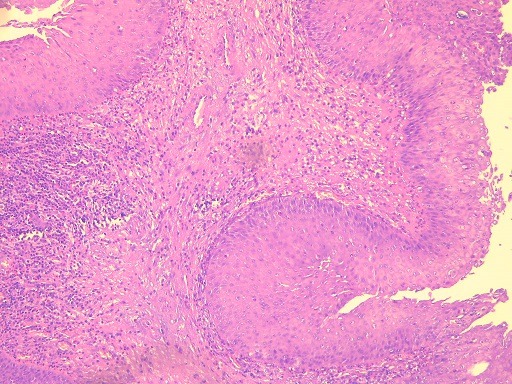
Microscopic view showing papillae and epithelial invaginations, sometimes covered by a transitional type epithelium sometimes of keratinized squamous type (HE x 100)

A 59-year-old patient consulted us for hearing loss, tinnitus and left otorrhea for one year. The patient had no dizziness or otalgia. He did not have a history of sinonasal pathology or surgery. Otoscopy revealed posterior perforation filled by irregular budding neoformation. The latter bleeds on contact. The contralateral ear was healthy. The rhinological examination was normal. The audiogram was in favor of a left hearing deafness of 50 db. The temporal CT scan showed tissue filling the tympanic cavity as well as the mastoid antrum without bone lysis (Figure 1). Magnetic resonance imaging (MRI) revealed a tissue process occupying the left middle ear in T1 and T2 isosignal and taking the contrast medium (Figure 2, Figure 3, Figure 4). The patient had a closed technique tympanoplasty. Perioperatively, the tympanic cavity and the mastoid were filled with polypoid tissue bleeding on contact. The orifice of the Eustachian tube was free. There is no bone lysis. An intraoperative biopsy of this polypoid tissue was made and the anatomopathological examination returned to the criteria corresponding to an inverted papilloma with papillae and epithelial invaginations, sometimes covered by a transitional type epithelium sometimes of keratinized squamous type. There were no signs of malignancy (Figure 5, Figure 6). Computed tomography of the facial plate in search of nasolabial localization was normal. The evolution was marked by the installation of a left otorrhea 4 months after the first surgery. The control MRI was in favor of recurrence. The patient was reoperated. He had a tympanoplasty in open technique. There was no recurrence after 30 months of surveillance.

## Discussion

Papillomas are rare benign tumors arising at the level of the Schneiderian membrane that covers the sinonasal tract. They are still called schneiderian papillomas. There are 3 types of papilloma: inverted papilloma is the most common (62%) followed by exophytic papilloma (32%) and oncocytic (6%). The histological diagnosis of inverted papilloma is based on the discovery of epithelial masses invaginated in the underlying chorion: this typical aspect is at the origin of the term “inverted” attributed to this tumor. The inverted papilloma of the middle ear is exceptional. It was first described by Ston *et al* in 1987 and since then only 45 cases have been published in the literature [1-10]. Three theories have been proposed to explain the development of inverted papilloma in the middle ear: the first explains the presence of IP in the middle ear by direct extension or migration of tumor cells from a sinonasal IP through the Eustachian tube. This theory is in doubt since several cases of middle ear IP have been reported in the literature without there being a synchronous sinonasal localization or metachrone and with a Eustachian tube which is perfectly free. In our case, the sinonasal tract and the Eustachian tube were free clinically and radiologically and there was no history of IP. The second theory involves an embryonic migration of an ectopic Schneiderian epithelium into the middle ear. The third mechanism is chronic inflammation related to chronic suppurative otitis media. This chronic inflammation stimulates the development of a Schneiderian-type mucosa that causes IP. These theories allow us to classify the IP of the middle ear in primary and secondary according to the presence or not of a synchronous sinonasal localization. In our case, it is a primary IP. Only 23 cases of primary inverted papilloma have been reported in the literature [1-6, 9]. The role of HPV (type 6, 11, 16 and 18) is still controversial; as well as the role of certain sex hormones, especially progesterone [5, 6, 8].

Hearing loss and otorrhea are the most common symptoms. Tinnitus, otalgia, fullness of the ear, headache, and facial palsy are rare. Three otoscopic aspects have been described in the literature. It may be a complete tympanum but is repressed by a retro-tympanic neoformation. The second aspect is that of a tympanic perforation with a fund that is budding. The third aspect is that of an irregular and bleeding neoformation that fills the bottom of the external auditory canal. Some differential diagnoses may be referred to as middle ear adenoma, paraganglioma, cholesteatoma and endolymphatic sac adenocarcinoma. Only the histological examination that confirms the diagnosis of IP. IPs are characterized by a high risk of local recurrence after surgery. The recurrence rate is higher than in the sinonasal localization. This can be explained by the fact that complete surgical excision is more difficult in the middle ear than in large sinonasal cavities. Similarly, the rate of malignant transformation of middle ear IP is greater than that in sinonasal locations [6, 10]. In a review of Schaefer's literature, of the 29 cases of middle ear IP studied, 12 (41%) showed degeneration. The recidivism rate was 48%. It is 100% in the tympanoplasty with a simple excision. The rate of degeneration of sinonasal IP is 9% and the recurrence rate varies between 5% and 21% [10]. The treatment of middle ear IP is surgical but it is difficult to define a standard surgical technique. It is important to have a large and complete excision to avoid recurrence. In fact, incomplete excision is accompanied by a high rate of recurrence. Postoperative radiotherapy is indicated in forms associated with malignant transformation [8, 9]. The realization of MRI allows early detection of recurrences. It differentiates between postoperative inflammatory changes and recurrence with sensitivity and specificity better than CT.

## Conclusion

The middle ear IP is extremely rare. The clinical and radiological signs are not specific. Only the histological examination that confirms the diagnosis. It is characterized by a high rate of recidivism and malignant transformation. The treatment of middle ear IP is surgical but it is difficult to define a standard surgical technique. It is important to have a large and complete excision to avoid recurrence.

## Competing interests

The authors declare no competing interests.
